# Supplementary material for: Towards automatic home-based sleep apnea estimation using deep learning
Source: NPJ Digit Med. 2024 Jun 1;7:144. doi: 10.1038/s41746-024-01139-z (PMC11144223; doi:10.1038/s41746-024-01139-z)
Supplement: Supplementary file 1 — Supplementary Material: Towards automatic home-based sleep apnea estimation using deep learning [file 41746_2024_1139_MOESM1_ESM.pdf]

Supplementary Material: Towards automatic home-based sleep apnea estimation using deep learning

Gabriela Retamales[1,†], Marino E. Gavidia[1,†], Ben Bausch[1], Arthur N. Montanari[1,2], Andreas Husch[1], and Jorge Goncalves[1,3,*]

[1]Luxembourg Centre for Systems Biomedicine, University of Luxembourg, L-4367 Belvaux, Luxembourg
[2]Department of Physics and Astronomy, Northwestern University, Evanston, IL 60208, USA
[3]Department of Plant Sciences, Cambridge University, Cambridge CB2 3EA, United Kingdom
[†]Equal contributions
[*]Corresponding author: jmg77@cam.ac.uk

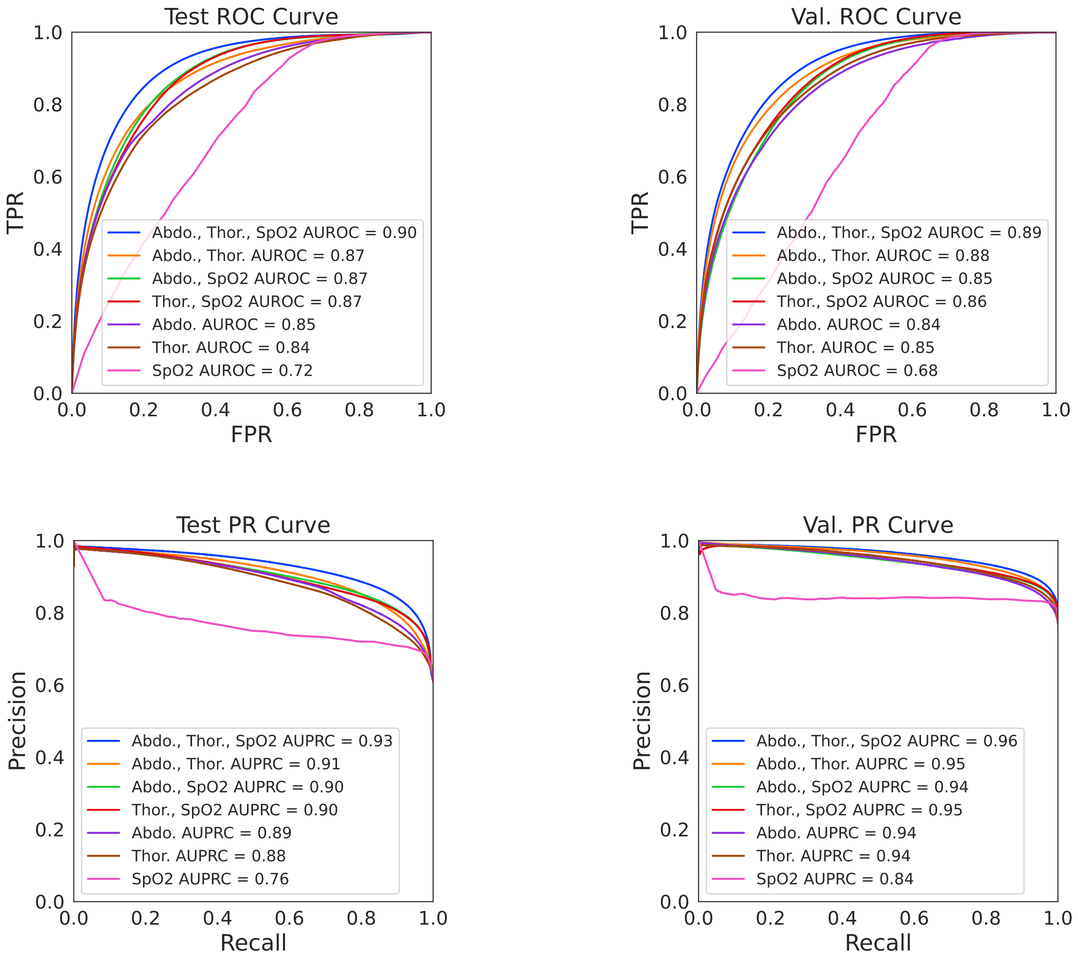

Supplementary Figure 1: Comparison between different sensor inputs to DRIVEN for sleep/awake detection on the test (left) and validation (right) datasets. The top and bottom panels show the ROC and PR curves, respectively.

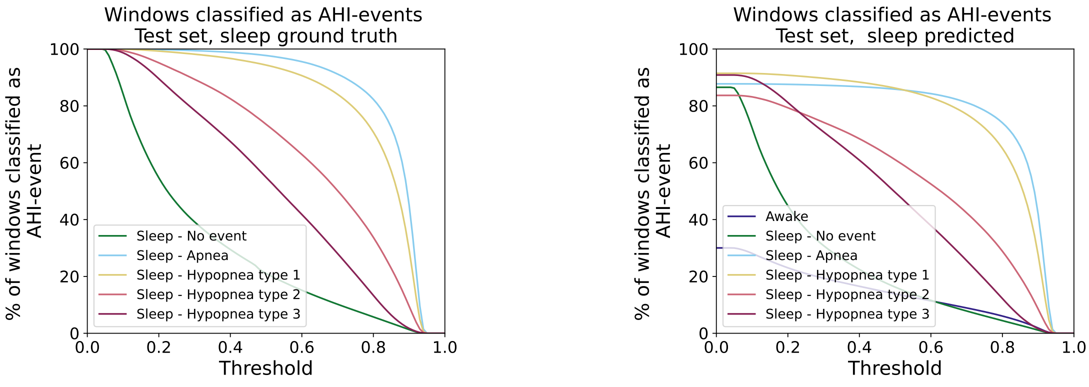

(a) (b)

Supplementary Figure 2: Percentage of 30 seconds windows classified as AHI-event per class on the test dataset. Each window is labeled from the profusion file as the following classes: awake, sleep without respiratory event, sleep with apnea, sleep with hypopnea type 1, sleep with hypopnea type 2, or sleep with hypopnea type 3. Each curve represents, as a function of the classification threshold, the percentage of events in the corresponding class classified as an AHI-event. The classification was performed using two input channels (abdominal movement and SpO_2). (a) Performance results are shown when the ground truth for sleep/awake events are used (therefore no "awake" is classified as an "AHI-event"). (b) Performance results are shown when sleep/awake detection is used (therefore some "awake" windows are classified as "sleep, no AHI-event" and some are classified as an "AHI-event"). In this case, certain "AHI-events" may be misclassified as "awake" events.

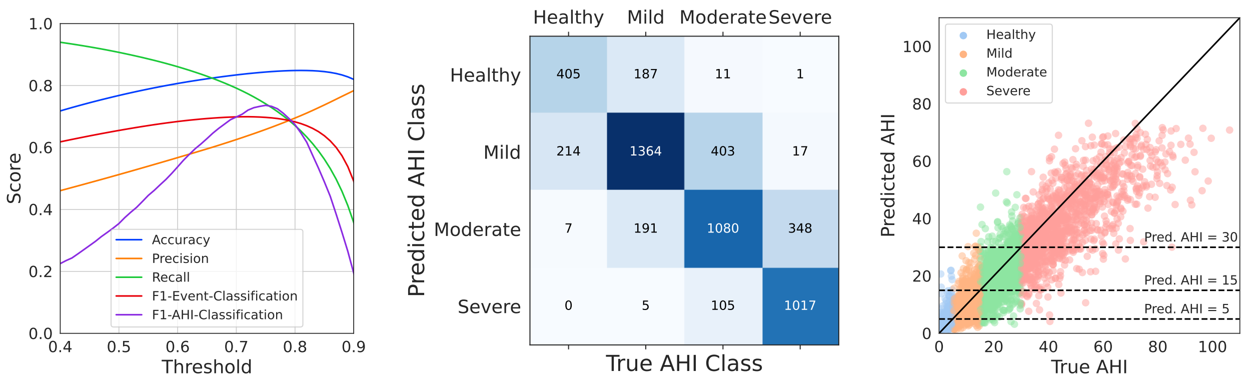

Supplementary Figure 3: Performance of DRIVEN on AHI estimation when using abdominal, thoracic, and SpO_2 sensors.

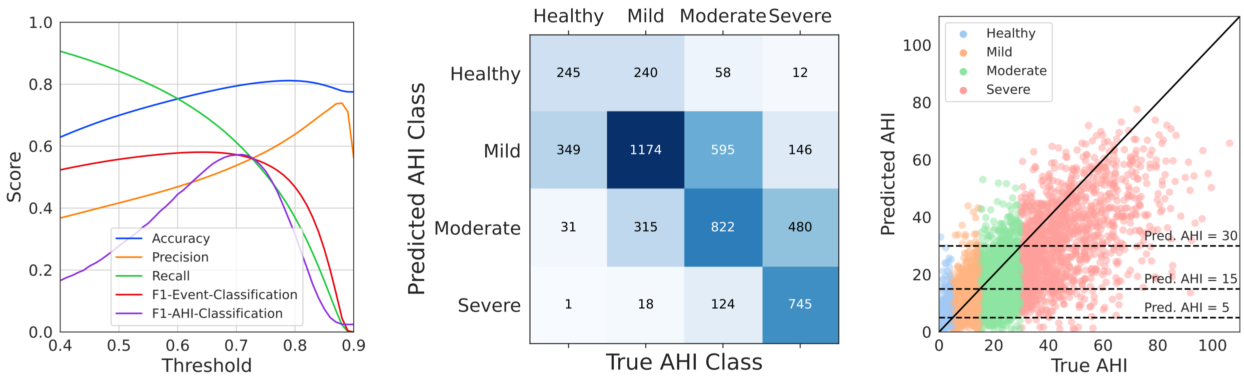

Supplementary Figure 4: Performance of DRIVEN on AHI estimation when using abdominal and thoracic sensors.

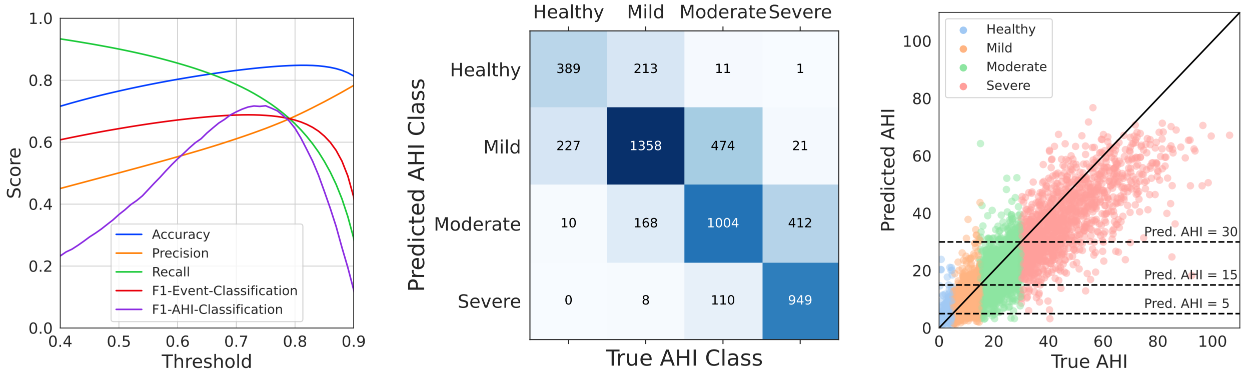

Supplementary Figure 5: Performance of DRIVEN on AHI estimation when using thoracic and SpO_2 sensors.

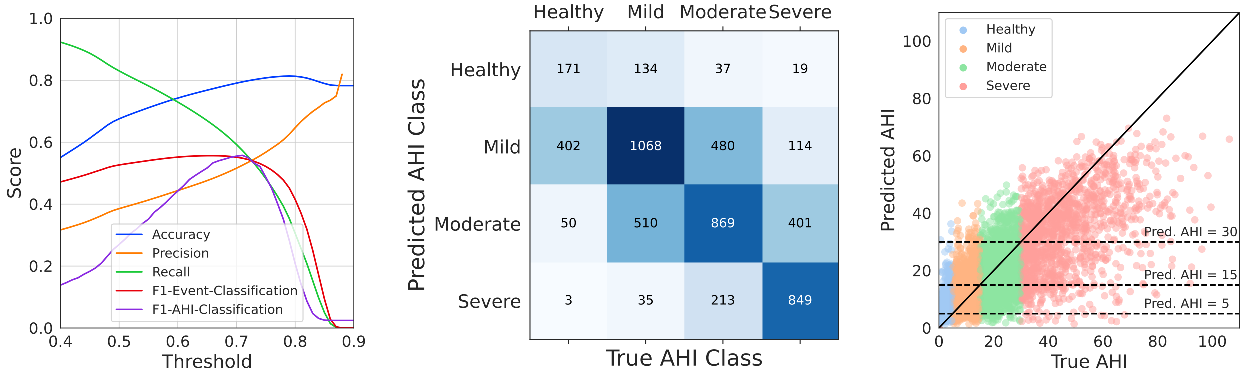

Supplementary Figure 6: Performance of DRIVEN on AHI estimation when using only the abdominal sensor.

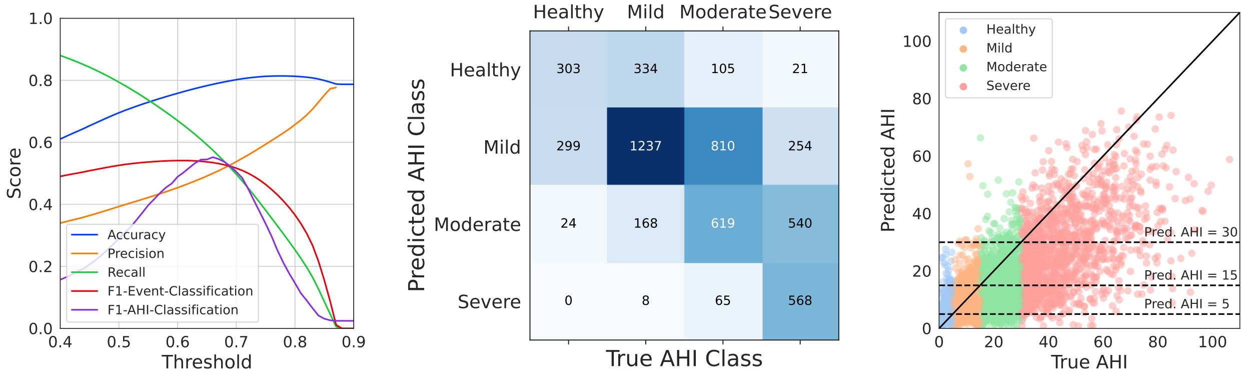

Supplementary Figure 7: Performance of DRIVEN on AHI estimation when using only the thoracic sensor.

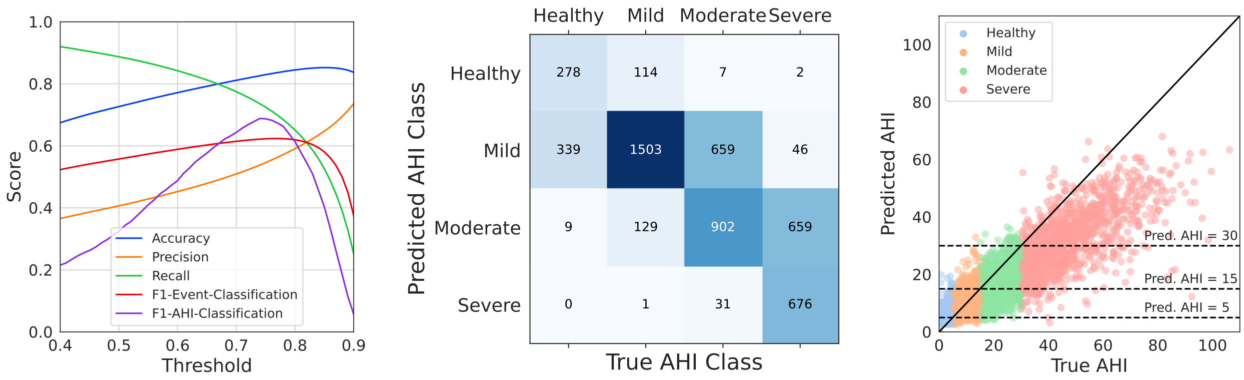

Supplementary Figure 8: Performance of DRIVEN on AHI estimation when using only the SpO_2 sensor.

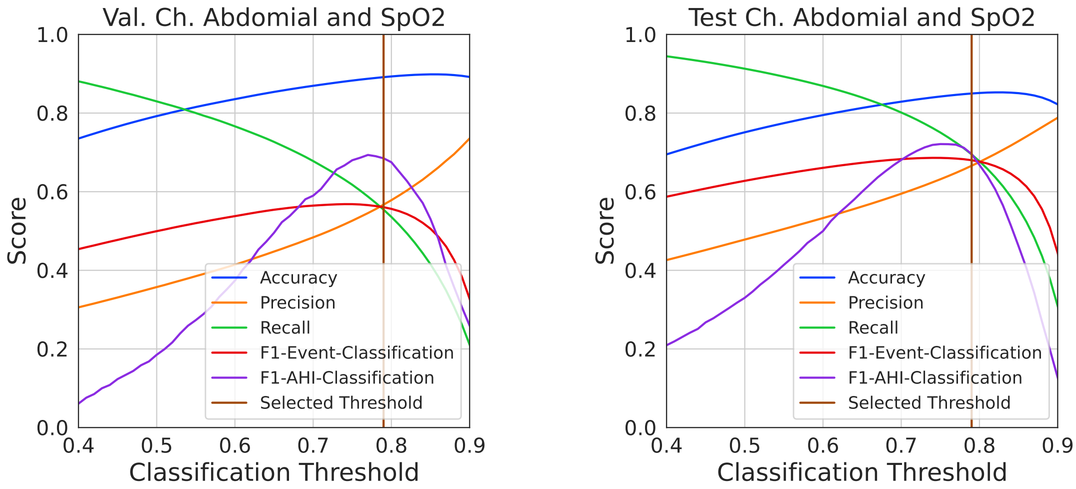

(a) (b)

Supplementary Figure 9: Choice of classification threshold. (a) The threshold used for classification was chosen as the point of intersection of the precision and recall curves on the *validation* dataset of trial 2. (b) Performance metrics for the *test* set, with the brown vertical line depicting the threshold chosen in panel (a).

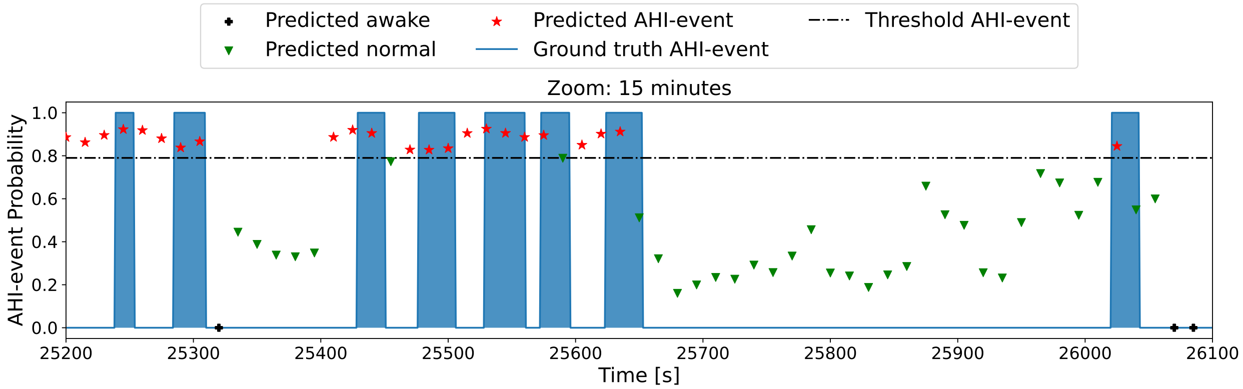

Supplementary Figure 10: Additional 15 minute zoom-in on a section of the data shown in Figure 4. It can be observed that detection is smoothed in-between AHI-events, which results in incorrectly detecting AHI-events between some individual events that closely follow each-other.

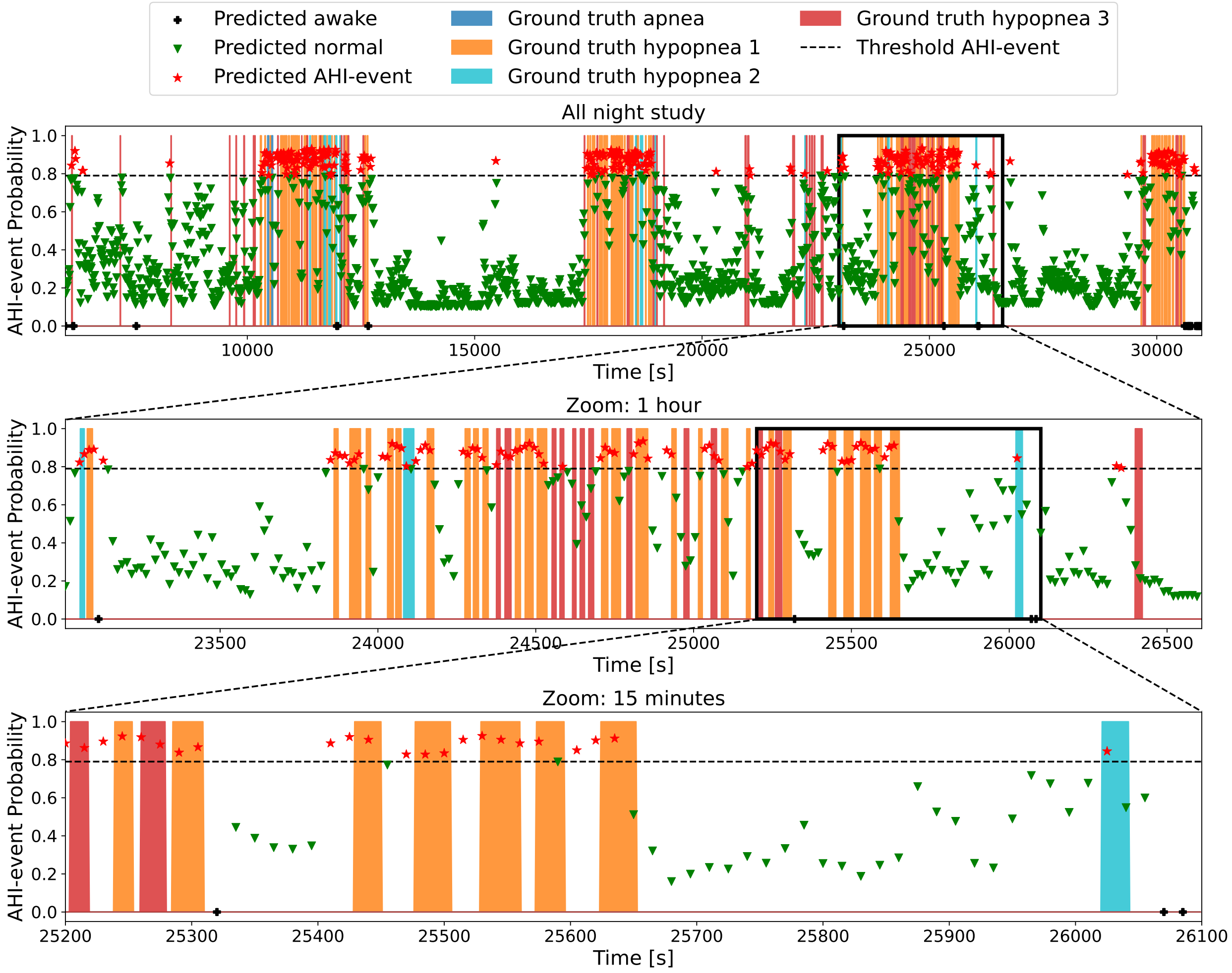

Supplementary Figure 11: Automatic labeling of AHI-events for the same patient as Figure 4 and Supplementary Figure 10 using two sensors (abdominal movement and SpO_2). The colored areas represent true events: blue for apnea events (flow reduction of more than 90%), orange for hypopnea type 1 (flow reduction of more than 30% and SpO_2 desaturation of more than 3%), cyan for hypopnea type 2 (flow reduction of more than 30% and arousal), and red for hypopnea type 3 (not considered in AHI). The output of DRIVEN is illustrated with symbols that represent, for each 30 second window, the probability of the window to be classified as an AHI-event. Finally, the windows are colored according to their classification, depending on whether they are above or below the determined threshold of 0.79. The black crosses represent the segments that were classified as awake, the green triangles the ones classified as not AHI-events, and the red stars are the windows classified as AHI-events. Going down, the second and third plots zoom in a segment of 1h and 15 minutes, respectively.

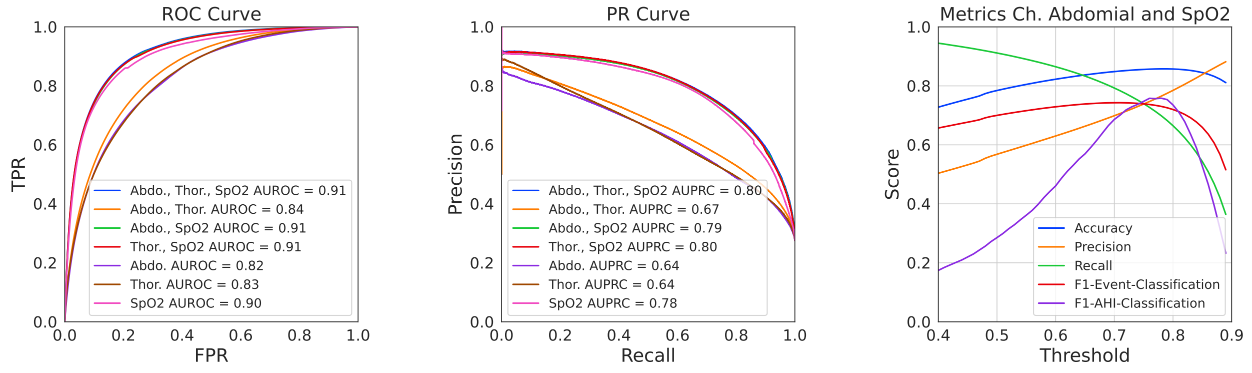

(a) (b) (c)

Supplementary Figure 12: Performance of DRIVEN on classification of AHI-events when ground-truth knowledge on the sleep/awake events is used for all patients in the test dataset. (a) Receiver-operator characteristic and (b) precision-recall curves. (c) Threshold dependent performance metrics for two input channels (abdominal movement and SpO_2).

Channels	Test		Validation	
	AUROC	AUPRC	AUROC	AUPRC
Abdominal + Thoracic + SpO_2 + RRI	0.900	0.924	0.893	0.958
Abdominal + Thoracic + SpO_2	0.901	0.925	0.894	0.959
Abdominal + Thoracic + RRI	0.887	0.915	0.888	0.957
Abdominal + Thoracic	0.871	0.906	0.878	0.954
Abdominal + SpO_2 + RRI	0.878	0.907	0.859	0.944
Abdominal + SpO_2	0.873	0.903	0.854	0.942
Thoracic + SpO_2 + RRI	0.871	0.902	0.867	0.947
Thoracic + SpO_2	0.869	0.901	0.863	0.946
Abdominal + RRI	0.866	0.898	0.855	0.943
Abdominal	0.850	0.890	0.842	0.940
Thoracic + RRI	0.857	0.892	0.864	0.947
Thoracic	0.839	0.884	0.854	0.944
SpO_2 + RRI	0.758	0.797	0.730	0.870
SpO_2	0.716	0.758	0.678	0.841
RRI	0.702	0.751	0.705	0.860

Supplementary Table 1: Comparison between different sensor inputs to DRIVEN and its measured AUROC and AUPRC scores for sleep/awake detection on the test and validation datasets in trial 2. The corresponding curves are shown in Supplementary Figure 1.

Channels	F1 patient AHI classification		
	No sleep information	Sleep ground truth	Predicted sleep
Abdominal, Thoracic, SpO_2	0.65	0.76	0.72
Abdominal, SpO_2	0.66	0.76	0.72
Thoracic, SpO_2	0.64	0.75	0.69
Abdominal, Thoracic	0.51	0.58	0.56
SpO_2	0.64	0.75	0.63
Abdominal	0.54	0.59	0.55
Thoracic	0.47	0.53	0.51

Supplementary Table 2: DRIVEN performance for different channel combinations when using no sleep information, corrected with PSG-labeled sleep state, and corrected with DRIVEN's sleep prediction.

Channels	Window Size					
	10		30		80	
	AUROC	AUPRC	AUROC	AUPRC	AUROC	AUPRC
Abdominal + Thoracic + Airflow + SpO_2 + RRI	0.899	0.593	0.921	0.719	0.923	0.793
Abdominal + Thoracic + Airflow + SpO_2	0.897	0.587	0.916	0.702	0.921	0.786
Abdominal + Thoracic + SpO_2 + RRI	0.889	0.563	0.914	0.697	0.920	0.786
Abdominal + Thoracic + SpO_2	0.888	0.561	0.913	0.690	0.914	0.770
Abdominal + Airflow + SpO_2 + RRI	0.890	0.578	0.911	0.693	0.916	0.777
Abdominal + Airflow + SpO_2	0.886	0.567	0.913	0.698	0.916	0.779
Thoracic + Airflow + SpO_2 + RRI	0.892	0.575	0.911	0.681	0.915	0.772
Thoracic + Airflow + SpO_2	0.890	0.567	0.913	0.693	0.917	0.780
Abdominal + Thoracic + Airflow + RRI	0.846	0.464	0.886	0.635	0.899	0.738
Abdominal + Thoracic + Airflow	0.841	0.461	0.884	0.626	0.904	0.750
Abdominal + SpO_2 + RRI	0.881	0.556	0.908	0.682	0.912	0.771
Abdominal + SpO_2	0.880	0.556	0.906	0.678	0.910	0.768
Thoracic + SpO_2 + RRI	0.878	0.535	0.906	0.669	0.902	0.735
Thoracic + SpO_2	0.878	0.532	0.903	0.659	0.909	0.751
Airflow + SpO_2 + RRI	0.877	0.551	0.901	0.668	0.903	0.755
Airflow + SpO_2	0.875	0.549	0.902	0.678	0.901	0.752
Thoracic + Airflow + RRI	0.831	0.419	0.880	0.613	0.890	0.724
Thoracic + Airflow	0.825	0.416	0.873	0.605	0.886	0.717
Abdominal + Airflow + RRI	0.832	0.440	0.875	0.609	0.894	0.735
Abdominal + Airflow	0.827	0.436	0.876	0.615	0.897	0.744
Abdominal + Thoracic + RRI	0.814	0.394	0.868	0.587	0.893	0.728
Abdominal + Thoracic	0.816	0.407	0.866	0.589	0.890	0.731
SpO_2 + RRI	0.846	0.484	0.877	0.603	0.878	0.683
SpO_2	0.850	0.487	0.875	0.605	0.871	0.676
Abdominal + RRI	0.789	0.353	0.852	0.561	0.881	0.714
Abdominal	0.779	0.357	0.846	0.569	0.872	0.711
Airflow + RRI	0.785	0.346	0.836	0.543	0.850	0.660
Airflow	0.760	0.337	0.829	0.539	0.844	0.664
Thoracic + RRI	0.772	0.299	0.833	0.505	0.855	0.643
Thoracic	0.769	0.296	0.834	0.517	0.857	0.659
RRI	0.576	0.134	0.621	0.230	0.663	0.326

Supplementary Table 3: Comparison of LGBM-3 model performance on AHI-events classification based on different sensor combinations and CNN input window sizes in a 3-fold cross-validation experiment with 1000 randomly selected patients (800 for training, 200 for validation). This is a summary table of the complete experiment provided in Supplementary Table 4.

Supplementary Table 4: Spreadsheet of classification performance on AHI-events classification for validation and test set of different evaluated models for a subset of 1,000 patients. Comparison of the LGBM-3, LGBM-1 and end-to-end CNN models for different sensor combinations (abdominal, thoracic, airflow, SpO_2 and RRI) and CNN input window sizes (10, 30 and 80 seconds) in a 3-fold cross-validation experiment with 1000 randomly selected patients (800 for training, 200 for validation). This table is included as a separate spreadsheet file.

Supplementary Table 5: Spreadsheet of classification performance on AHI-events classification and AHI estimation for validation and test set for three complete data splits, with pre selected models from Supplementary Table 4. Comparison of the LGBM-3 and LGBM-1 different sensor combinations (abdominal, thoracic, airflow and SpO_2) and CNN input window sizes (30 and 80 seconds). This table is included as a separate spreadsheet file.
